# Fascia Iliaca Compartment Block for Perioperative Pain Management of Geriatric Patients with Hip Fractures: A Systematic Review of Randomized Controlled Trials

**DOI:** 10.1155/2020/8503963

**Published:** 2020-11-25

**Authors:** Hao-yang Wan, Su-yi Li, Wei Ji, Bin Yu, Nan Jiang

**Affiliations:** ^1^Division of Orthopaedics & Traumatology, Department of Orthopaedics, Nanfang Hospital, Southern Medical University, Guangzhou 510515, China; ^2^Department of Medical Quality Management, Nanfang Hospital, Southern Medical University, Guangzhou 510515, China; ^3^Division of Spine Surgery, Department of Orthopaedics, Nanfang Hospital, Southern Medical University, Guangzhou 510515, China

## Abstract

**Background:**

With continuous increase of the aging population, the number of geriatric patients with fragility hip fractures is rising sharply, and timely surgery remains the mainstay of treatment. However, adequate and effective pain control is the precondition of satisfactory efficacy. This systematic review aimed to summarize the use of fascia iliaca compartment block (FICB) as an analgesic strategy for perioperative pain management in geriatric patients with hip fractures.

**Methods:**

PubMed and Embase databases were searched for English published randomized controlled trials (RCTs) reporting application of FICB for pain control of the older adults with hip fractures between January 1^st^, 2000, and May 31^st^, 2020. The modified Jadad scale was used to evaluate quality of the RCTs included. Primary outcomes of the eligible RCTs were presented and discussed.

**Results:**

A total of 27 RCTs with 2478 cases were included finally. The present outcomes suggested, after admission or in the emergency department (ED), FICB can provide patients with equal or even better pain relief compared with the conventional analgesia methods, which can also reduce additional analgesic consumptions. While, before positioning for spinal anesthesia (SA), FICB is able to offer superior pain control, facilitating SA performance, after surgery FICB can effectively alleviate pain with decreased use of additional analgesics, promoting earlier mobilization and preventing complications.

**Conclusions:**

FICB is a safe, reliable, and easy-to-conduct technique, which is able to provide adequate pain relief during perioperative management of geriatric patients with hip fractures.

## 1. Introduction

Hip fracture, an important and debilitating condition in the older adults, represents a worldwide challenge [1]. With the progressive aging population, hip fracture has become a significant public health issue worldwide. It is estimated the absolute number of hip fractures is expected to increase from 1.6 million in 2000 to 6.3 million by the year 2050 [2]. Besides, hip fracture ranks among the top 10 of disability [3]. Furthermore, the increasing number of patients with hip fractures and the great risk of limb disability aggravate the economic burdens, both personally and socially. The estimated annual cost of hip fracture treatment in the US had increased from approximate 10.3 to 15.2 billion dollars in 1990 [4] to 17 billion in 2002 [5]. Nowadays, hip fracture has become a widely concerning social problem.

Geriatric patients with hip fractures without adequate pain control are reluctant to mobilize, thus increasing the potential risk of complications and slows the recovery [6]. It is known that cognitive impairment has been widely reported in geriatric patients with hip fractures, which is partly attributed to the untreated or not-well-controlled pain. A previous study indicated that cognitively intact patients with hip fractures with untreated pain were nine times more likely to develop delirium than those with effective pain control [7]. Thus, adequate analgesia is of great significance. In addition, patients with hip fractures are at high risks of many complications, and even some are fatal, such as pneumonia [8], pressure ulcer [9], urinary tract infection [10], and deep venous thrombosis [11]. Hence, timely surgery remains the mainstay of treatment [12]. In order to achieve satisfying clinical efficacy and lower the risk of adverse events, appropriate analgesic methods play a vital role.

Current strategies for pain management include oral and parenteral systemic analgesia, such as paracetamol, nonsteroidal anti-inflammatory drugs (NSAIDs), opioids, epidural and spinal anesthesia (SA), and peripheral nerve blocks [13]. Although opioids have been widely applied, they can provide effectively static pain relief, but they may be inadequate for dynamic pain [14]. Besides, opioids may bring side effects, especially for the older adults, such as delirium, drowsiness, constipation, nausea, and even respiratory depression, which may affect prognosis of the patients [15–17]. In order to lower the risk of adverse events and also guarantee the treatment efficacy, different analgesia strategies have been investigated and compared.

Considering the particularity of this cohort, recently, peripheral nerve blockade or regional anesthesia has become an increasingly attractive option in delivering effective pain relief, with fascia iliaca compartment block (FICB) as a representative. FICB or fascia iliaca block (FIB), first proposed by Dalens et al. in 1989, is a means of blocking the three principal lumbar plexus nerves of the thigh with a single injection of local anesthetic delivered immediately dorsal to the fascia iliaca [18, 19]. Indications of FICB are surgical anesthesia to the lower extremity, management of cancer pain and pain owing to inflammatory conditions of the lumbar plexus, and amelioration of acute pain following trauma, fracture, and burn [20], while contraindications of FICB are few, including patients with coagulopathy, those who are taking antithrombotic medications, infection at the injection site, or history of femoral bypass surgery [21, 22]. Besides, allergies to the anesthetic agents and crush injury at or near the injection site are set as absolute contraindications [21, 22].

Recently, as the number of studies investigating the use of FICB as a new analgesia strategy in the treatment of geriatric patients with hip fractures is rising, it is necessary to summarize current experience of this technique. Here, we conducted a systematic review aiming to review the present knowledge regarding the use of FICB for pain management during perioperative treatment of geriatric patients with hip fractures.

## 2. Methods

Literature search was performed by two independent authors in the PubMed and Embase databases to identify English published randomized controlled trials (RCTs) regarding the use of FICB as a pain relief strategy in perioperative management of geriatric patients with hip fractures between January 1^st^, 2000, and May 31^st^, 2020. The following search strategy was used: “(hip fracture OR femur fracture) AND (Fascia iliaca block OR Fascia iliaca nerve block OR Fascia iliaca compartment block OR Fascia iliaca compartment nerve block OR Fascia iliac block OR Fascia iliac nerve block OR Fascia iliac compartment block OR Fascia iliac compartment nerve block OR FICB OR FIC OR FIB).”

Only RCTs evaluating the application of FICB for perioperative pain management in geriatric patients with hip fractures were considered. Exclusion criteria were FICB applied in nonhip fracture or nongeriatric patients. In addition, studies that did not provide adequate information for quality assessment or data analysis were also excluded.

Two authors independently screened the titles, abstracts, and even full texts to make sure that the retrieved RCTs should strictly meet the inclusion criteria. Two authors independently evaluated the quality of the RCTs included using the modified Jadad scale [23], an eight-item scale designed to assess randomization, blinding, withdrawals and dropouts, inclusion and exclusion criteria, adverse effects, and statistical analysis (Table 1). The score for each study could range from 0 (lowest quality) to 8 (highest quality). Scores of 4 to 8 denote good to excellent quality and 0 to 3 poor to low quality. Disagreement was resolved by discussion, and if necessary, a third author's opinion was consulted for final decision.

## 3. Results

**Figure 1:**
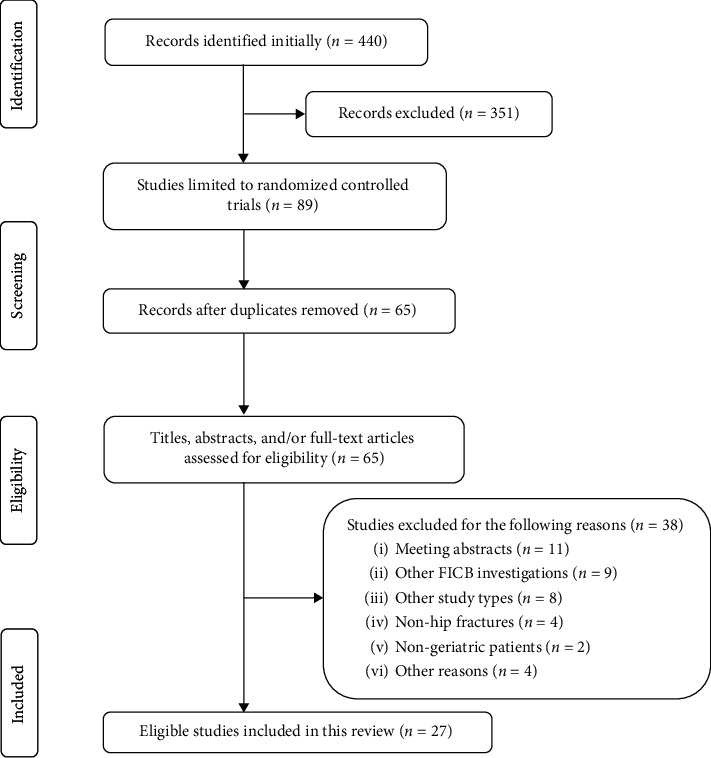
Preferred reporting items for systematic reviews and meta-analyses flow diagram.

Altogether, 440 publications were identified initially. After limiting study type to RCTs, removing the duplicates, screening the titles, and evaluating the abstracts and/or full texts, we finally included 27 RCTs studies [24–50] with 2478 cases. The eligibility selection process is shown in Figure 1.

According to the modified Jadad scale, 26 RCTs were rated as good (Table 2). However, only two studies [33, 44] gave a clear description on the method used to assess adverse effects. In addition, 9 studies [25, 30, 40–44, 46, 49] were not designed as blinded. Although another 5 studies [24, 29, 36, 39, 50] were designed as blinded, they did not describe the blinding method in detail.

The RCTs included were mainly divided into three groups according to the stage of FICB use and the primary outcomes reported, including preoperative use (12 RCTs), application before surgical anesthesia (4 RCTs), and postoperative use (8 RCTs).  Table 3 provides general information, comparison details, FICB strategy, primary outcome parameters, and conclusions of the RCTs included. The visual analog scale (VAS) pain score, additional analgesia use, and adverse effects were the most frequently reported outcome measures.

## 4. Discussion

### 4.1. FICB for Pain Management before Surgery

During the acute phase following hip fractures, it is essential and important to provide geriatric patients with adequate pain relief, which assists them in moving about in bed, using a bedpan, and receiving preoperative preparations [24]. The frequently used methods for pain relief include NSAIDs and opioids, while NSAIDs increase potential risk of bleeding and can exacerbate underlying gastrointestinal problems in geriatric patients. Improper opioids use may also cause a high risk of adverse events, such as hypotension, sedation, and even respiratory depression [51]. How to balance between adequate pain control and minimum risk of adverse events remains a great challenge. Recent RCTs reported the efficacy of using FICB technique for preoperative pain management in geriatric patients with hip fractures (Table 3).

Outcomes of several RCTs indicated that the analgesic effect of FICB is better than that of the opioids. A 2007 RCT [24] compared the efficacy of FICB with intramuscular injection of 0.1 mg/kg morphine in patients suspected of hip fracture before radiograph test in the emergency department (ED). Outcomes showed patients who received FICB achieved maximum pain relief both at rest and on movement, with a significantly less morphine consumption, and a decreased proportion of patients who required sedation. In a subsequent 2015 RCT, McRae et al. [25] compared FICB with intravenous morphine and also obtained better efficacy after FICB disposition, without immediate adverse events. These outcomes demonstrate that FICB may provide better pain control in hip fracture than morphine, administered either intramuscularly or intravenously. However, the sample sizes of the two studies are limited. In addition to the comparisons between FICB and morphine, two RCTs also investigated potential efficacy of FICB as an adjuvant therapy to routine preoperative analgesics (e.g., morphine and paracetamol); however, their conclusions differed [26, 27]. Wennberg et al. [26] concluded that low-dose FICB was an effective pain-relieving adjuvant to other analgesics, while Pasquier et al. [27] failed to find any significant effect of FICB as an adjuvant therapy. Their different conclusions may be associated with several possible factors. First, FICB strategies including anesthesia types and concentrations differed between the two studies. Second, outcome measures and detection time points were also different. Third, different sample sizes may also influence the outcomes, especially for the study by Pasquier et al. [27], which only included 15 participants for each group. It also should be noted that in the study by Wennberg et al. [26], aside from morphine, paracetamol was also applied; the single use of paracetamol in controlled group may be another source for explanation of different conclusions between the two studies.

NSAIDs are first-line analgesics as an alternative to opioids, and recent studies also compared the analgesic effect of FICB with NSAIDs. A 2010 RCT [28] showed that the mean VAS score of patients at 15 min following NSAIDs injection was significantly lower than those by FICB. However, the scores of patients who received FICB at 2 h and 8 h were lower than those who received NSAIDs, despite no statistical differences. They concluded that FICB is nearly as effective for up to about 8 h after administration and can effectively control post-hip fracture pain, with a rapid onset. Later in 2018, Ma et al. [29] evaluated the use of FICB in the very older adults (over 80 years) with hip fractures, with a traditional method (50 mg tramadol plus 500 mg paracetamol, orally, three times a day) set as controls. Outcomes revealed that the VAS pain scores under different phases in patients who had received FICB were significantly lower than those of the controls, including scores at rest and in the morning of the day of surgery, as well as passive movement scores at 1 h after analgesia at the time of admission and in the morning of the day of surgery. Aside from RCTs, a non-RCT also indicated the definite efficacy of FICB as an effective pain relief strategy for patients with proximal femur fractures, as compared with NSAIDs [52].

In addition to the comparisons of FICB with opioids and NSAIDs, previous RCTs also compared efficacy of FICB with other different analgesic methods achieved by local injections, including femoral nerve block (FNB), “3-in-1” block, and even intra-articular hip injection (IAHI). In an RCT published in 2013, Newman et al. [30] performed comparisons between FICB and FNB guided by nerve stimulator in patients with femoral neck fractures. Outcomes revealed that patients who underwent FNB had better analgesic effect than those who received FICB, with less morphine consumption following FNB. Similarly, although outcomes of a 2019 RCT revealed that both FICB and FNB were effective in pain control, patients managed by FNB showed better analgesic efficacy, with lower incidences of nausea and vertigo [31]. However, another 2019 RCT did not find significant difference regarding the reduction in pain scores between FICB and FNB, suggesting their similar efficacy [32]. Several factors may account for the differed outcomes, such as drug dose and concentration, experience of the physicians, and detection points as well. As for the “3-in-1” block, it was first described by Winnie et al. in 1973 [53] and shares similarities with FICB, as both are single-injection anterior thigh approach techniques aiming at blocking the femoral, obturator, and lateral femoral cutaneous nerves [33]. Outcomes of a 2015 RCT revealed similar efficacy between the two techniques in relieving the immediate pain following femur fractures [33]. Apart from FNB and “3-in-1” block, even a study evaluated the efficacy of FICB versus IAHI. In this RCT, Aprato et al. [34] found better efficacy following IAHI treatment, with less supplement of systemic analgesia. However, considering many possible confounding factors, such as the limited number of such reports and safety and handleability of this technique, more future studies are necessary.

It is known that impaired cognition is one of the major risk factors for perioperative delirium in geriatric patients with hip fractures. A recent double-blind RCT [35] investigated the effects of preoperative FICB use on cognition. However, they failed to find a positive association between preoperative pain relief by FICB and the cognition status of the included patients. Considering a low-dose FICB administered as a supplement to regular analgesia in this study, this discrimination requires to be addressed in future studies.

As mentioned previously, feasibility of technique conducting is also of great importance, especially in the ED. In fact, conducting of FICB does not require complicated equipment or assistance and even can be performed by junior doctors [16] and trained paramedics [25], which greatly improves the efficiency in the ED and pre-hospital settings. Høgh et al. [54] analyzed the efficacy of FICB technique performed by junior registrars (JR) in preoperative pain management for patients with hip fractures. Outcomes demonstrated that FICB performed by JR is feasible, which requires minimal introduction and no expensive equipment and is connected with a minimal risk approach. Similarly, a recent study also conveyed that conducting FICB by junior doctors and specialist nurses in the ED is feasible and safe and improves the proportion of patients receiving blocks [55].

In general, most RCTs found that FICB displays better analgesic effect than opioids and NSAIDs. However, controversy exists with regard to the comparisons of FICB with other nerve block techniques. Despite this issue, FICB has been confirmed to be a feasible, safe technique, with most patients achieving satisfactory efficacy in pain relief prior to surgery.

### 4.2. FICB as an Adjuvant to Surgical Anesthesia

It is known that, for geriatric patients with hip fractures, spinal anesthesia (SA) is a widely accepted anesthetic strategy, which reveals a lower mortality and lower risks of adverse events compared with general anesthesia [56]. However, positioning for SA is a great challenge for both patients and anesthesiologists as movement is extremely painful, resulting in major patient distress. In addition, inadequate pain relief may cause physiological sequelae, such as tachycardia, hypertension, and increased cardiac work that may compromise high-risk cardiac patients [37]. Therefore, it is important to conduct effective management of pain, not only for patient comfort, but also for easier performance of the central nervous blockades. Recent RCTs investigated the efficacy of FICB as an adjuvant to surgical anesthesia (Table 3).

In a 2009 RCT, Yun et al. [36] compared the efficacy of FICB with a continuous infusion of alfentanil prior to SA for geriatric patients with femoral neck fracture. Outcomes revealed that patients who received FICB had a lower mean VAS score during positioning and a shorter mean time to achieve SA, with better patient acceptance than the controls. Later in 2014, another RCT [37] evaluated FICB versus intravenous fentanyl for positioning hip fracture patients for SA; aside from the above parameters, they also found that FICB implementation was associated with a lower morphine consumption after surgery and a longer duration to the first dose demand. In a 2016 RCT, Madabushi et al. [38] once again confirmed the superiorities of FICB before positioning for SA; in addition to the above issues, they also reported significantly improved sitting angle in FICB group. A recent double-blinded RCT [39] found that the mean total duration of analgesia after SA predisposed with FICB was significantly longer, which may help explain the previous findings that patients who received FICB had a lower morphine consumption and a longer duration to the first dose requirement [37].

In short, although the number of RCTs reporting FICB as an adjuvant prior to SA remains limited, current RCTs, based on different outcome parameters, suggested that conducting FICB before positioning for SA in geriatric patients with hip fractures can provide superior pain management compared with traditional methods, facilitating SA performance, yielding satisfactory postoperative analgesia with wide acceptance, thus improving the overall quality and efficiency of care [37].

### 4.3. FICB for Pain Management after Surgery

After hip surgery, adequate pain relief is also important, which can facilitate earlier mobilization, restore limb function, and prevent complications. Recent RCTs also assessed the efficacy of FICB in postoperative pain management from different perspectives (Table 3).

A 2014 RCT compared the postoperative analgesia effect of FNB with FICB in patients with hip fractures, and outcomes showed better efficacy following FNB intervention, with a lower amount of additional analgesia and a lower rate of side effects [40]. It is reasonable to understand these outcomes, as in this study FNB was provided continuously, whereas FICB was performed only once. At the same year, another RCT compared FICB with 3-in-1 block for postoperative pain control in patients who received prosthesis surgery as a result of hip fracture. Results showed similar efficacy of FICB and 3-in-1 block, also with similar tramadol consumption between the two [41]. A 2016 prospective RCT [42] evaluated the efficacy of FICB after hemiarthroplasty, and outcomes revealed a significant opioid-sparing effect in the first 24 h after surgery with FICB supplement. Later in 2018, an RCT [43] compared the effect of patient-controlled FICB with patient-controlled intravenous fentanyl (PC-IVF) for pain management after surgery. Outcomes showed satisfactory efficacy following FICB with decreased additional analgesia use and side effects. Later in 2019, Yamamoto et al. evaluated the effect of FICB versus intravenous acetaminophen on improvement of postoperative pain on movement, and they concluded that FICB achieved better efficacy without increasing the risk of complications [44]. However, they also indicated that no significant differences were found between the two regarding the total number of rescue analgesics required and the time to first standing. A new 2020 RCT showed that patients who received preoperative FICB had a statistically reduced postoperative morphine consumption and an increased proportion in patient-reported satisfaction [45], which is also supported by another 2020 RCT [46]. Aside from the definite efficacy of FICB in the alleviation of postoperative acute pain, it may also play an active role in the relief of chronic postsurgical pain (CPSP). Diakomi et al. [47] examined the impact of FICB on the development of CPSP after hip surgery, and they found that FICB group presented with lower hip-related characteristic pain intensity scores at 3 months postoperatively, with a lower percentage of patients with high-grade CPSP at 3 and 6 months after surgery. This investigation is novel and interesting, which implies that FICB may also have a positive effect on CPSP. Considering that only one RCT addressed this issue, more future studies are necessary.

In summary, although the outcome measures differed, most RCTs revealed benefits of FICB in postoperative pain relief. However, the number of such RCTs is few, with limited sample size; therefore, more future RCTs are warranted to more comprehensively assess the effect of FICB in postoperative pain management.

### 4.4. Other Benefits of FICB Application in Geriatric Patients with Hip Fractures

In addition to the above advantages, FICB technique may also bring other benefits in geriatric patients with hip fractures. Many studies reported that the use of FICB could reduce the risk of perioperative complications, such as delirium, pruritus, nausea, and vomiting [48–50], decrease the length of hospital stay, and accelerate functional recovery [29, 57, 58] (Table 3).

In a 2009 RCT, Mouzopoulos et al. [48] investigated the prophylaxis of FICB on perioperative delirium, and the patients included were divided into three different groups based on delirium risk (low, intermediate, and high). Although the prophylactic effect of FICB on high-risk patients was not obvious, it significantly decreased the incidence of delirium in patients in an intermediate risk. Thus, they concluded that FICB may be beneficial for perioperative delirium, especially for those in intermediate risk. Subsequently, a 2015 RCT [49] indicated the definite efficacy of FICB in alleviating postoperative pain, together with lower rates of postoperative nausea and vomiting (PONV) and pruritus, as compared with the patient-controlled intravenous analgesia (PCIA) using fentanyl. Interestingly, they observed a higher incidence of postoperative delirium in FICB group, implying that, aside from pain, many other factors may also influence the occurrence of delirium. In a recent double-blind RCT, Hao et al. [50] indicated that preoperative continuous FICB use was effective in reducing the risk of postoperative delirium. In addition to RCTs, a recently published meta-analysis [59], comprising 11 RCTs with 937 patients, indicated that FICB could reduce the total consumption of morphine and the incidence of nausea. One of the possible explanations for the decreased risk of delirium following FICB may be attributed to the reduced supplementary analgesics. However, as mentioned above, occurrence of delirium is affected by multiple factors, especially in such a cohort at a higher risk to develop delirium.

Aside from RCTs, still other studies evaluated the influence of FICB on cognitive performance. Callear and Shah [60] found that the rate of patients who had experienced postoperative delirium following SA was twice that by FICB. In a synthesis analysis of 21 RCTs assessing the efficacy of additional peripheral nerve blockade for hip fracture surgery, Rashiq et al. [61] compared FICB, FNB, and lumbar and sacral plexus block in prophylaxis of delirium. Outcomes showed that FICB had the highest probability of being the most effective against delirium. In a retrospective study comprising 959 patients aged over 65 years with a femoral neck fracture, Odor et al. [62] investigated potential influence of FICB on postoperative abbreviated mental test scores (AMTS). Outcomes revealed that FICB use at admission was linked to significantly higher adjusted odds for a higher AMTS relative to lower AMTS than conventional analgesia method. Thus, they suggested that FICB use at patient admission may help improve early postoperative cognitive performance.

In addition to the decreased risk of complications, FICB can also help reduce the length of hospital stay and accelerate the functional recovery. A previous RCT [29] reported that the mean length of hospital stay in patients that received FICB was significantly shorter than that of the controls. Similarly, Lees et al. [57] found the acute length of hospital stays in patients managed by FICB decreased to an average of 9.9 days, compared with 15 days of the control group. Similar outcome was also found in another pilot study [58]. Moreover, even one study [57] reported that the inpatient mortality in the FICB group was statistically lower than that in the control group (5.5% vs. 15%), whereas another one failed to find any statistical significance [63]. Of course, it should be noted that the mortality of such cohort of patients is influenced by multiple factors apart from analgesic methods, such as age, underlying disease, comorbidity, and treatment strategy. Therefore, cautious attitude should be taken towards the results.

### 4.5. Limitations and Future Perspectives

Although, in recent years, the number of RCTs investigating FICB as an analgesic strategy in the treatment of geriatric patients with hip fractures is rising, the sample size of most studies is limited. Thus, the outcomes and conclusions should be interpreted with caution. Then, the outcome parameters reported by different studies varied, making it more difficult to draw a conclusion with consistent results. Moreover, the detailed strategy of FICB (e.g., anesthetic type and dose, and interval between FICB and SA) as well as the control group settings also differed from each other, rendering it unavailable for data synthesis analysis. Although several systematic reviews and meta-analyses tried to sum up findings from published RCTs, such a high heterogeneity among RCTs may lead to a higher risk of bias.

Therefore, in order to achieve more accurate and reliable conclusions, high-quality RCTs with a larger sample size are essential. In addition, the standard reporting items of FICB investigation may be established, and if possible, standard FICB procedure should be considered. Furthermore, in-depth analyses should be performed to optimize the application of FICB in pain management in the older adults with hip fractures.

## 5. Summary

Growing evidence suggests that FICB is an effective and reliable strategy for preoperative pain relief in geriatric patients with hip fractures. After admission or at the ED, FICB use can provide adequate pain control, which can also decrease additional analgesics consumption. Prior to positioning for SA, FICB can facilitate conducting SA, yield satisfactory postoperative analgesia, and improve the overall quality and efficiency of care, while after surgery FICB can also provide adequate pain relief with decreased supplementary analgesics, promoting earlier hip mobilization, restoring limb function, and preventing postoperative complications. In the future, more high-quality RCTs should be conducted to more comprehensively evaluate and optimize the FICB technique for perioperative pain management in geriatric patients with hip fractures.

## Figures and Tables

**Table 1:** The modified Jadad scale with eight items.

Item assessed	Response	Score
Was the study described as randomized?	Yes	+1
	No	0
Was the method of randomization appropriate?	Yes	+1
	No	−1
	Not described	0
Was the study described as blinded?^*∗*^	Yes	+1
	No	0
Was the method of blinding appropriate?	Yes	+1
	No	−1
	Not described	0
Was there a description of withdrawals and dropouts?	Yes	+1
	No	0
Was there a clear description of the inclusion/exclusion criteria?	Yes	+1
	No	0
Was the method used to assess adverse effects described?	Yes	+1
	No	0
Was the method of statistical analysis described?	Yes	+1
	No	0

^*∗*^Double-blind obtains 1 score; single-blind obtains 0.5 score.

**Table 2:** Methodological assessment of the RCTs included.

Study	Random description	Random method	Blinding description	Blinding method	Withdrawals/dropouts description	Inclusion/exclusion criteria	Adverse effects assessment	Statistical methods description	Total score
Foss et al. 2007 [24]	Yes	Yes	Yes	ND	Yes	Yes	No	Yes	6
McRae et al.2015 [25]	Yes	Yes	No	ND	Yes	Yes	No	Yes	5
Wennberg et al. 2019 [26]	Yes	Yes	Yes	Yes	Yes	Yes	No	Yes	7
Pasquier et al. 2019 [27]	Yes	Yes	Yes	Yes	Yes	Yes	No	Yes	7
Godoy Monzón et al. 2010 [28]	Yes	Yes	Yes	Yes	Yes	Yes	No	Yes	7
Ma et al. 2018 [29]	Yes	Yes	Yes	ND	Yes	Yes	No	Yes	5.5
Newman et al. 2013 [30]	Yes	Yes	No	ND	Yes	Yes	No	Yes	5
Zhou et al. 2019 [31]	Yes	Yes	Yes	Yes	No	Yes	No	Yes	6
Cooper et al. 2019 [32]	Yes	Yes	Yes	Yes	Yes	Yes	No	No	6
Reavley et al. 2015 [33]	Yes	Yes	Yes	Yes	Yes	Yes	Yes	Yes	7.5
Aprato et al. 2018 [34]	Yes	Yes	Yes	Yes	Yes	Yes	No	Yes	6.5
Wennberg et al. 2019 [35]	Yes	Yes	Yes	Yes	Yes	Yes	No	Yes	7
Yun et al. 2009 [36]	Yes	Yes	Yes	ND	No	Yes	No	Yes	4.5
Diakomi et al. 2014 [37]	Yes	Yes	Yes	Yes	Yes	Yes	No	Yes	6.5
Madabushi et al. 2016 [38]	Yes	Yes	Yes	Yes	No	Yes	No	Yes	5.5
Kacha et al. 2018 [39]	Yes	Yes	Yes	ND	No	Yes	No	Yes	5
Temelkovska-Stevanovska et al. 2014 [40]	Yes	ND	No	ND	No	Yes	No	Yes	3
Deniz et al. 2014 [41]	Yes	ND	No	ND	Yes	Yes	No	Yes	4
Bang et al. 2016 [42]	Yes	Yes	No	ND	Yes	Yes	No	Yes	5
Mostafa et al. 2018 [43]	Yes	Yes	No	ND	Yes	Yes	No	Yes	5
Yamamoto et al. 2019 [44]	Yes	Yes	No	ND	Yes	Yes	Yes	Yes	6
Thompson et al. 2020 [45]	Yes	Yes	Yes	Yes	Yes	Yes	No	Yes	6.5
Schulte et al. 2020 [46]	Yes	Yes	No	ND	Yes	Yes	No	Yes	5
Diakomi et al. 2020 [47]	Yes	Yes	Yes	Yes	Yes	Yes	No	Yes	7
Mouzopoulos et al. 2009 [48]	Yes	Yes	Yes	Yes	Yes	Yes	No	Yes	6.5
Nie et al. 2015 [49]	Yes	Yes	No	ND	Yes	Yes	No	Yes	5
Hao et al. 2019 [50]	Yes	Yes	Yes	ND	Yes	Yes	No	Yes	5.5

ND: not described.

**Table 3:** RCTs evaluating FICB in pain management in geriatric patients with hip fracture.

Study	Country	Comparison and no. of the included patients	FICB strategy	Outcome parameters	Primary conclusions
Preoperative use					
Foss et al. 2007 [24]	Denmark	FICB = 24 vs IM morphine = 24	40 mL 1.0% mepivacaine	VRS (rest/movement), total morphine consumption	FICB provided better pain relief at all times and at all measurements compared to IM morphine
McRae et al.2015 [25]	Australia	FICB = 11 vs standard care (IV morphine) = 13	15–20 mL 2% lidocaine, weight-dependent	NRS, adverse events	FICB group had a greater reduction in pain than those who received standard care
Wennberg et al. 2019 [26]	Sweden	FICB = 66 vs placebo (saline) = 61 (adjunctive therapy)	30 mL 0.2% ropivacaine	VAS	Low-dose FICB improved pain management as a pain-relieving adjuvant to other analgesics
Pasquier et al. 2019 [27]	Switzerland	FICB = 15 vs placebo (saline) = 15 (adjunctive therapy)	30 mL 0.5% bupivacaine	NRS (rest/movement), total morphine consumption	Anatomic landmark-based FICB did not help reduce pain after prehospital morphine
Godoy Monzón et al. 2010 [28]	Argentina	FICB = 62 vs IV NSAIDs (Diclofenac or Ketorolac) = 92	0.3 mL/kg 0.25% bupivacaine	VAS	FICB can provide equally effective analgesia as NSAIDs for up to 8 h
Ma et al. 2018 [29]	China	CFICB = 44 vs oral drugs (tramadol and paracetamol) = 44	50 mL 0.4% ropivacaine, 5 mL/h 0.2% ropivacaine continuously	VAS (rest/movement), patients' satisfaction, side effects, length of hospital stay	Patients treated with CFICB received better analgesia both at rest and at movement compared to traditional analgesia
Newman et al. 2013 [30]	UK	FICB = 56 vs FNB = 51	20–30 mL 0.5% levobupivacaine, weight-dependent	VAS, opioid consumption	Patients treated with FNB had better pain control and less morphine requirement
Zhou et al. 2019 [31]	China	FICB = 77 vs FONB = 77	35 mL 0.4% ropivacaine	VAS (rest/exercise), requirement for analgesic drugs, postoperative complications	Both FONB and FICB were effective in acute pain control. FONB performed better in reducing pain and function recovery
Cooper et al. 2019 [32]	Australia	FICB = 52 vs FNB = 48	20 mL 0.5% levobupivacaine	NRS	FICB can provide equivalent analgesia effect as FNB for femur fracture patients
Reavley et al. 2015 [33]	UK	FICB = 88 vs “3-in-1”block = 90	2 mg/kg 0.5% bupivacaine	VAS	FICB was as effective as “3-in-1” block for immediate pain relief
Aprato et al. 2018 [34]	Italy	FICB = 70 vs IAHI = 50	40 mL 0.25% ropivacaine	NRS (rest/movement), additional analgesic drug, adverse events	IAHI provided better pain management and reduced systemic analgesia consumption compared with FICB
Wennberg et al. 2019 [35]	Sweden	FICB = 65 vs control = 60	30 mL 2 mg/mL ropivacaine	Changes of cognitive status	FICB did not affect cognitive status in this study

Application before surgical anesthesia					
Yun et al. 2009 [36]	Korea	FICB = 20 vs IV alfentanil = 20	30 mL 0.375% ropivacaine	Time to achieve SA, VAS, quality of patient positioning, patient acceptance	FICB was more efficacious than IV alfentanil with better pain control during positioning and shorter time to achieve SA as well
Diakomi et al. 2014 [37]	Greece	FICB = 21 vs IV fentanyl = 20	40 mL 0.5% ropivacaine	Time needed and quality of position, NRS, postoperative analgesia, morphine consumption, patient satisfaction	Patients who received FICB showed significantly lower pain score, shorter spinal performance time, and better quality of position
Madabushi et al. 2016 [38]	India	FICB = 30 vs IV fentanyl = 30	30 mL 0.375% ropivacaine	VAS, sitting angle, positioning quality, time to perform SA, postoperative analgesic requirement	Patients who received FICB needed less time for SA and had better quality of positioning accompanied by superior analgesia
Kacha et al. 2018 [39]	India	FICB = 50 vs placebo (normal saline) = 50	30 mL 0.25% ropivacaine	VAS, time of positioning SA, total duration of analgesia	FICB effectively provided analgesia during positioning for SA and significantly extended the total duration of analgesia

Postoperative use					
Temelkovska-Stevanovska et al. 2014 [40]	Macedonia	FICB = 30 vs FNB = 30	40 mL 0.25% bupivacaine	VDS (rest/movement), additional analgesia, and duration for the first time, side effects	FNB provided superior postoperative pain relief versus FICB, and lower amount of supplemental analgesia
Deniz et al. 2014 [41]	Turkey	FICB = 20 vs “3-in-1” block = 20 vs control = 20	30 mL 0.25% bupivacaine	VAS, opioid consumption, adverse effects, and cortisol and ACTH levels	Both FICB and “3-in-1” block can bring superior analgesia and reduction in opioid consumption. The two blocks also showed a suppression of stress hormones
Bang et al. 2016 [42]	Korea	FICB = 11 vs. Non-FICB = 11	40 mL 0.2% ropivacaine	Postoperative VAS scores, opioid consumption, and adverse events	The FICB had a significant opioid-sparing effect in the first 24 hours after hemiarthroplasty
Mostafa et al. 2018 [43]	Egypt	FICA = 30 vs. IV fentanyl = 30	35 mL 0.125% levobupivacaine + PC-FICA^*∗*^	Postoperative VAS scores, additional analgesia requirement, and total additional analgesia assumption	PC-FICA provided a better quality of analgesia and decreased postoperative rescue analgesic requirement without increased side effects compared to PCA IV fentanyl
Yamamoto et al. 2019 [44]	Japan	FICB = 25 vs IV acetaminophen = 28	40 mL 0.25% levobupivacaine	VAS (rest/movement), total number of rescue analgesics required, incidence of delirium	Patients treated with FICB received better pain control compared to IV NSAIDs without increasing the complication rate
Thompson et al. 2020 [45]	America	FICB = 23 vs control = 24	30 mL 0.25% ropivacaine	Pain medication consumption, functional recovery, patient satisfaction	FICB significantly decreased postoperative consumption of morphine for breakthrough pain while increasing patient satisfaction
Schulte et al. 2020 [46]	USA	FICB = 57 vs control = 40	45 to 60 mL 0.375% ropivacaine	VAS, MME, postoperative ambulatory distance	A single perioperative FIB for patients with hip fractures undergoing surgery may decrease opioid consumption and increase the likelihood that a patient is discharged home
Diakomi et al. 2020 [47]	Greece	FICB = 91 vs sham FICB = 91	40 mL 0.5% ropivacaine	Incidence, intensity, and severity of CPSP at 3 and 6 months after hip fracture surgery	FICB in the perioperative setting may reduce the incidence, intensity, and severity of CPSP at 3 and 6 months after hip fracture surgery, providing safe and effective postoperative analgesia

Other benefits of FICB					
Mouzopoulos et al. 2009 [48]	Greece	FICB = 102 vs placebo (water for injection) = 105	0.25 mg dose of 0.3 mL/kg bupivacaine	Perioperative delirium, mean duration of delirium	Severity and incidence of delirium were significantly lower in intermediate-risk patients treated with FICB, along with shorter mean duration of delirium
Nie et al. 2015 [49]	China	CFICB = 51 vs PCIA (IV fentanyl) = 53	20–30 mL 0.5% ropivacaine, 0.1 mL/kg/h 0.25% ropivacaine continuously	Postoperative pain and complications (delirium, nausea and vomiting, and pruritus)	FICB showed a stronger effect on reducing postoperative nausea and vomiting, and pruritus, but with a higher incidence of developing delirium
Hao et al. 2019 [50]	China	CFICB = 44 vs placebo (normal saline) = 46	30 mL 0.45% ropivacaine, 6 mL/h 0.25% ropivacaine continuously	Postoperative delirium, change in preoperative and postoperative pain scores, opioid consumption	The incidence of post-op delirium was lower for patients who received CFICB

RCTs: randomized controlled trials; FICB: fascia iliaca compartment block; VRS: verbal rating scale; IM: intramuscular; IV: intravenous; NRS: numerical rating scale; VAS: visual analogue scale; NSAIDs: non-steroidal anti-inflammatory drugs; CFICB: continuous fascia iliaca compartment block; FNB: femoral nerve block; FONB: femoral obturator nerve block; IAHI: intra-articular hip injection; SA: spinal anesthesia; VDS: verbal descriptive scale; ACTH: adrenocorticotropic hormone; PCIA: patient-controlled intravenous analgesia; FICA: fascia iliaca compartment analgesia; PC-FICA:: patient-controlled fascia iliaca compartment analgesia; MME: morphine milligram equivalents; CPSP: chronic postsurgical pain. ^*∗*^Protocol: a continuous basal infusion of 4 mL/h levobupivacaine 0.125% and demand boluses of 2 ml with a lockout interval of 15 min.

## Data Availability

The data used to support the findings of this systematic review are from previously published studies, which have been cited.
